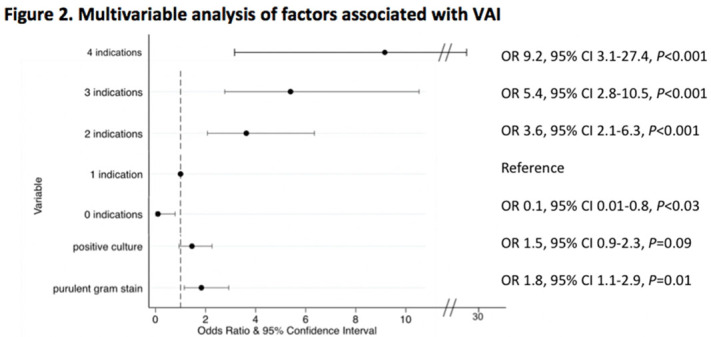# Indications for and Utility of Tracheal Aspirate Cultures for the Diagnosis of VAI

**DOI:** 10.1017/ash.2021.116

**Published:** 2021-07-29

**Authors:** Kathleen Chiotos, Giyoung Lee, Guy Sydney, Heather Wolfe, Jennifer Blumenthal, Hannah Stinson, Julie Harab, Danielle Traynor, Joseph Piccione, Ashlee Doll, Garrett Keim, Charlotte Woods-Hill, Megan Jennings, Rebecca Harris, Jeffrey Gerber, Aaditya Dudhia, Nancy McGowan, Jennalyn Burke

## Abstract

**Background:** Tracheal aspirate bacterial cultures are routinely collected in mechanically ventilated children for the evaluation of ventilator-associated infections (VAIs). However, frequent bacterial colonization of endotracheal and tracheostomy tubes contribute to the marginal performance characteristics of the test for diagnosing VAI. Published literature characterizing drivers of culture collection and the predictive value of positive cultures are limited. **Methods:** This single-center, retrospective cohort study included children admitted to the pediatric intensive care unit who were receiving mechanical ventilation for at least 48 hours and had 1 or more semiquantitative tracheal aspirate cultures collected between September 1, 2019, and August 31, 2020. Indications for culture collection were determined through medical record review and included fever, hypothermia, tracheal secretion changes, radiographic pneumonia, increased oxygen requirement, and/or increased positive end-expiratory pressure (PEEP). A positive culture was defined as moderate or heavy growth of a noncommensal bacterial organism. A purulent Gram stain was defined as detection of moderate or many white blood cells. Diagnosis of VAI was based on treating-clinician documentation and was ascertained through medical record review. Logistic regression accounting for clustering by patient was performed to estimate the association between indications for culture collection and (1) culture positivity, (2) purulent Gram stain, and (3) diagnosis of VAI. **Results:** In total, 625 tracheal aspirate cultures were performed in 261 unique patients. Common indications for culture collection included isolated fever or hypothermia (n = 124, 20%), fever with an increase in oxygen requirement or PEEP (n = 71, 11%), isolated increase in oxygen requirement or PEEP (n = 67, 11%), or isolated secretion change (n = 54, 9%) (Figure 1). Overall, 230 cultures (37%) were positive and 218 (35%) Gram stains were purulent. There were no associations between culture indications and a positive culture. Presence of isolated fever was negatively associated with a purulent Gram stain (odds ratio [OR], 0.49; 95% CI, 0.30–0.81; *P* = .005); otherwise, there were no associations between indication and purulent Gram stain. Finally, in a multivariable model, odds of VAI diagnosis increased with both the number of indications for culture collection and purulent Gram stain, but not with positive culture (Figure 2). **Conclusions:** Number and type of clinical signs were not associated with tracheal aspirate culture positivity or purulence on Gram stain, but they were associated with a clinical diagnosis of VAI. These findings suggest that positive tracheal aspirate cultures may not aid clinicians in the diagnosis of VAI, and they highlight the opportunity for improved diagnostic stewardship.

**Funding:** No

**Disclosures:** None

**Figure 1.**
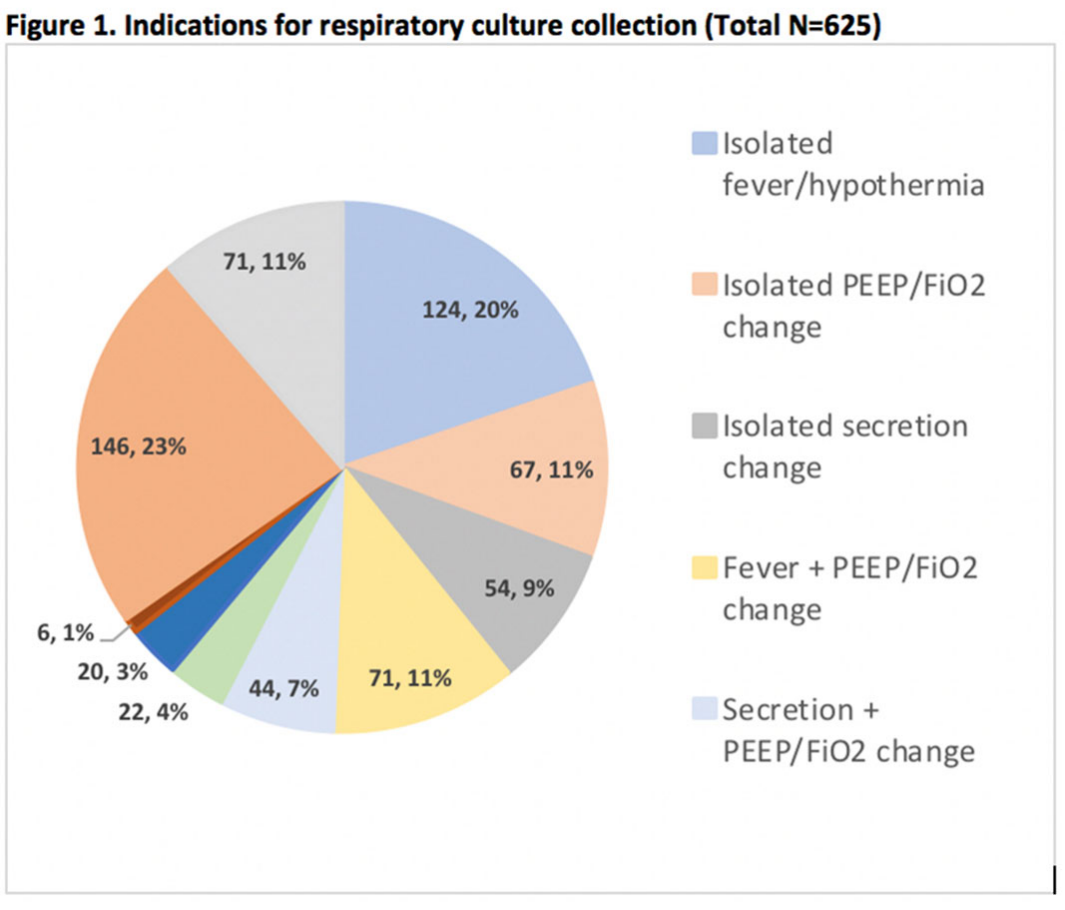

**Figure 2.**